# Paid Leave Mandates and Care for Older Parents

**DOI:** 10.1111/1468-0009.12708

**Published:** 2024-06-20

**Authors:** KANIKA ARORA, DOUGLAS A. WOLF

**Affiliations:** ^1^ University of Iowa; ^2^ Maxwell School of Citizenship and Public Affairs Syracuse University

**Keywords:** family caregivers, paid leave

## Abstract

**Policy Points**

- We examined the effect of the Paid Family Leave policy (PFL) and Paid Sick Leave policy (PSL) on care provision to older parents.
- We found that PSL adoption led to an increase in care provision, an effect mainly attributable to respondents in states/periods when PSL and PFL were concurrently offered. Some of the strongest effects were found among women and unpartnered adult children.
- PFL adoption by itself was not associated with care provision to parents except when PFL also offered job protection.
- Paid leave policies have heterogeneous effects on eldercare and their design and implementation should be carefully considered.

**Context:**

Family caregivers play a critical role in the American long‐term care system. However, care responsibilities are known to potentially conflict with paid work, as about half of family caregivers are employed. The federal Family and Medical Leave Act passed by the US Congress in 1993 provides a nonuniversal, unpaid work benefit. In response, several states and localities have adopted the Paid Family Leave policy (PFL) and Paid Sick Leave policy (PSL) over the last two decades. Our objective is to examine the effect of these policies on the probability of personal care provision to older parents.

**Methods:**

This study used longitudinal data from the Health and Retirement Study (1998‐2020). Difference‐in‐differences regression models were estimated to examine associations between state‐ and local‐level PFL and PSL mandates and personal care provision to older parents. We analyzed heterogeneous effects by the type of paid leave exposure (provision of job protection with PFL and availability of both PSL and PFL [with or without job protection] concurrently). We also examined results for different population subgroups.

**Findings:**

PSL implementation was associated with a four‐ to five‐percentage point increase in the probability of personal care provision. These effects were mainly attributable to respondents in states/periods when PSL and PFL were concurrently offered. The strongest effects were found among adult children who were employed at baseline, women, younger, unpartnered, and college educated. PFL implementation by itself was not associated with care provision to parents except when the policy also offered job protection.

**Conclusions:**

Paid leave policies have heterogeneous impacts on personal care provision, potentially owing to differences in program features, variation in caregiving needs, and respondent characteristics. Overall, the results indicate that offering paid sick leave and paid family leave, when combined with job protection, could support potential family caregivers.

Family members provide the majority of hands‐on care to community‐dwelling older adults living with disability or with serious health conditions.^1^ Although caregiving is not a new role, the share of family caregivers facing simultaneous demands of full‐time employment and increasingly complex caregiving responsibilities, extending over months or even years, has grown substantially in recent decades.^2^

The trade‐off between work and eldercare is well documented. About half of those caring for older individuals are employed.^1^ Intensive care provision, particularly by women, reduces work hours,^3^ increases the probability of labor market exits, and fosters both absenteeism (caregiving‐related work absences) and presenteeism (reduced productivity while at work).^4^, ^5^ Survey data show that a substantial number of employed caregivers make informal arrangements (such as arriving late or leaving early) or take sick or vacation time to accommodate caregiving demands.^6^, ^7^ Analogously, working additional hours per week reduces the probability of care provision and parental coresidence among women of prime caregiving ages.^8^, ^9^

The Paid Family Leave policy (PFL) and Paid Sick Leave policy (PSL)—two similar, yet distinct, workplace policies—may support individuals juggling competing responsibilities of work and eldercare by providing paid time off for family caregiving. Currently, the United States does not provide a statutory federal entitlement to either PFL or PSL. According to the Bureau of Labor Statistics, only 24% of private sector employees have PFL through their employers. Although PSL is more common, as many as 25% of workers do not have access to even a single paid sick day.^10^

Absent a federal requirement, some states and localities have enacted PFL and PSL programs for eligible employees experiencing qualifying family or health events.^11^ California passed the first statewide PFL law in 2004; since then, 11 states and Washington, DC, have mandated PFL on a statewide basis.^12^ The first PSL law was passed in 2006 in San Francisco by voter initiative. Since then, 14 states (including Washington, DC) and 23 cities and counties require employers to provide PSL coverage to employees.^13^

Although efforts to advance paid leave have gained momentum at national and state levels—most recently with the passage of the Families First Coronavirus Response Act (FFCRA), a temporary federal mandate for paid sick and family leave during the COVID‐19 pandemic—there is limited evidence to guide policy formulation targeting working Americans trying to reconcile eldercare and workplace commitments. Prior research has mainly assessed outcomes for new parents and their children resulting from PFL access (e.g., parental employment, breastfeeding practice, and infant health), employee health and health care use resulting from PSL mandates (e.g., contagious illnesses, injuries, and vaccination uptake), or firm‐level outcomes resulting from mandating either policy (e.g., productivity, other nonmandated fringe benefits, and labor costs).^14^, ^15^, ^16^, ^17^, ^18^, ^19^, ^20^, ^21^, ^22^, ^23^, ^24^, ^25^, ^26^, ^27^, ^28^, ^29^, ^30^, ^31^, ^32^, ^33^, ^34^

Only a few papers have analyzed the impact of paid leave policies on long‐term care patterns and, in particular, on outcomes for caregivers who are *not* new parents. One study showed that California's PFL program decreased nursing home utilization among older adults.^35^ Others demonstrated that PFL increased employment among individuals residing with a family member in poor health.^36^, ^37^, ^38^ A recent paper found that after PFL passage, women living in close proximity to parents in poor health were more likely to work while providing care and less likely to be depressed.^39^ However, a qualitative examination of caregivers’ experiences with PFL in California and New Jersey showed that lack of awareness, cumbersome application procedures, a lack of buy‐in from employers, and issues with benefit design comprised significant barriers to the uptake of paid family leave in these states.^40^ No prior work has examined the impact of PSL on family caregiving to older adults, although one recent study estimated the impact of PSL laws on caregiving leaves, measured as missing work because of either “childcare problems” or a generic category of “family/personal obligations.”^41^

Using data collected in the Health and Retirement Study (HRS)’s 1998–2020 interviews, we examined the effect of state‐mandated and locally mandated paid leave policies on the provision of personal care to one's parents. In doing so, we make three contributions to the literature. First, although past research has typically focused on the impact of either PFL or PSL alone, we examined the effect of PFL *and* PSL policies, both individually and in combination, on the probability of personal care provision to older parents among individuals in midlife who have at least one living parent. Second, we investigated the distinction between PFL laws that do and those that do not include a guarantee of job protection. We also studied the heterogeneous effects of both policies according to respondent characteristics. Third, several PSL studies examine the effect of state‐level mandates while disregarding policies passed by local jurisdictions. Similarly, much of the evidence on PFL impacts is based on California's first‐in‐the‐nation program. In this paper, we account for all paid leave laws implemented prior to 2021, including local jurisdictions in the case of PSL, while going beyond California to include the PFL policies of New Jersey, New York, and Washington.

## Background and Conceptual Framework

### Paid Leave Policies in the United States

With the expiration of FFCRA in 2020, the only federal policy that continues to allow workers time off for caregiving purposes is the Family and Medical Leave Act (FMLA) of 1993. Under the FMLA, eligible workers can take job‐protected leave of up to 12 weeks for the purposes of family caregiving. However, the FMLA's eligibility requirements (including job tenure, hours worked in the past year, and employer size) imply that roughly 40% of workers do not have access to the FMLA.^42^ Importantly, leave covered under the FMLA is *unpaid*. Lack of pay is a significant barrier to taking family leave. In a 2018 survey, two‐thirds of workers who did not take needed family and medical leave reported that they could not afford to take leave without pay.^43^ In the absence of more comprehensive federal programs, states and localities are increasingly establishing PFL and PSL programs to address health and family needs of workers.

Although both PFL and PSL provide paid time off for the purposes of caring for a family member, there exist important distinctions between the two programs. PFL typically provides workers with several weeks of paid time away from work to tend to their own or a family member's serious health condition or to bond with a new child. The maximum leave duration typically ranges from 6 to 12 weeks across mandating states.^12^ PFL programs allow workers to take leave on an intermittent basis. However, taking intermittent PFL may entail additional complexity because the leave schedule needs to be anticipated at the time of initial application, with limited scope for subsequent changes.^40^ PFL provides partial wage replacement (60%‐90% in most mandating states) with benefits capped at a maximum weekly amount.^12^ Not all state PFL mandates explicitly require employers to offer job protection. Workers in PFL states that do not offer job protection may take PFL simultaneously with the FMLA to gain employment protection while on leave.^44^ However, state PFL benefits typically cover a broader set of workers than the FMLA, which reduces the likelihood of being eligible for job‐protected PFL in these states.^12^ Leave under PFL typically requires a medical certification from a licensed health professional.^45^

PSL allows workers to accrue job‐protected time away from work, at full pay, to attend to their own short‐term health needs or to care for a family member. PSL offers leave on an accrual basis, usually taken intermittently in hourly or daily increments. In several PSL mandate states, employees can accrue up to 40–48 hours of paid sick leave a year, with usage generally limited to 40 hours.^13^ Unused hours often may be carried over to the next calendar year. PSL replaces 100% of a worker's regular wages with built‐in job‐protection benefits.^12^ Leave under PSL can be taken on short notice and typically does not require certification from a licensed health professional. Selected features of the laws included in this study are shown in Tables 1, 2, 3. The potential duration and procedural requirements, as well as the consequences for a worker's earnings, differ substantially from jurisdiction to jurisdiction.

**Table 1:** Characteristics of State and Local PFL and PSL Laws Included in the Analysis: State PFL Laws

State	Month/Year Benefits Implemented	Maximum Available, Weeks	Wage Replaced, %	Weekly Benefit Cap (2022), $	Job Protection^a^	Medical Certification Required?
CA	7/2004	8	60‐70^b^	1,357	No	Yes
NJ	7/2009	12	85^c^	993	No	Yes
NY	1/2018	12	67^c^	1,068	Yes	Yes
WA	1/2020	12	50‐100^c^, ^d^	1,327	No	Yes

PFL, Paid Family Leave policy; PSL, Paid Sick Leave policy.

This table was sourced from the Bipartisan Policy Center.^46^

^a^ “Yes” indicates that job protection is unconditional.

^b^ These percentages are applied to the worker's own earnings.

^c^ These percentages are applied to statewide average earnings.

^d^ The percentage depends on the worker's earnings.

**Table 2:** Characteristics of State and Local PFL and PSL Laws Included in the Analysis: State PSL Laws

State	Month/Year Benefits Implemented	Maximum Accrual Rate	Maximum Accrual	Within‐State Variation
Washington, DC	11/2008	1 per 37^a^	7 days	By firm size^b^
CA	7/2015	1 per 30	24 hours	No
MA	7/2015	1 per 30	40 hours	11+ employees only^c^
OR	1/2016	1 per 30	40 hours	10+ employees only
VT	1/2017	1 per 52	24 hours	By firm size (2017‐2018)
AZ	7/2017	1 per 30	40 hours	By firm size
WA	1/2018	1 per 40	40 hours	No
MD	2/2018	1 per 30	64 hours	15+ employees only
NJ	10/2018	1 per 30	40 hours	No
MI	3/2019	1 per 35	40 hours	50+ employees only
NV	1/2020	1 per 52	40 hours	50+ employees only

PFL, Paid Family Leave policy; PSL, Paid Sick Leave policy.

This table was sourced from the National Partnership for Women & Families.^12^

^a^ This is read as “1 hour accrued per __ hours worked.”

^b^ The accrual rate and/or maximum depend on firm size.

^c^ Smaller firms are not required to offer paid sick leave.

^d^ The maximum accruals depend on firm size.

**Table 3:** Characteristics of State and Local PFL and PSL Laws Included in the Analysis: Lower‐Level PSL Laws^2^, ^3^

Jurisdiction	Month/Year Benefits Implemented	Maximum Accrual Rate	Maximum Accrual, Hours	Within‐Jurisdiction Variation
San Francisco	2/2007	1 per 30^a^	40 (72)	Depends on firm size^b^
New York City (five counties)	4/2014	1 per 30	40	5+ employees only
Philadelphia	5/2015	1 per 40	40	10+ employees only^c^
Montgomery County, MD	10/2016	1 per 30	56	5+ employees only
Chicago	7/2017	1 per 40	40	No
Westchester County, NY	4/2019	1 per 30	40	5+ employees only

PFL, Paid Family Leave policy; PSL, Paid Sick Leave policy.

This table was sourced from the National Partnership for Women and Families.^13^ Several additional cities have also enacted a PSL mandate at some point. We included only counties in which the entire county, or cities whose population constitutes half or more of the county, has adopted a PSL law.

^a^ This is read as “1 hour accrued per __ hours worked.”

^b^ The accrual rate and/or maximum depend on firm size.

^c^ Smaller firms are not required to offer PSL.

### Family Care in the Context of Paid Leave Policies

Theoretically, both PFL and PSL mandates can be expected to benefit employed caregivers as both reduce the cost—in lost earnings—of taking leave. Therefore, both can be predicted to increase leave taking for caregiving purposes and, consequently, the prevalence or duration of caregiving activity. However, the responses to the two types of policies are likely to differ in important ways depending on caregiving needs and specific policy provisions that may influence access to and ease of taking family leave. We discuss these factors below.

Eldercare is a response to needs that are dynamic, heterogeneous, and often unpredictable.^1^ For individuals caring for older adults with chronic progressive conditions such as dementia, the caregiving role is likely to expand over time, with multiple periods of high‐intensity care that are sometimes punctuated with catastrophic events occurring with little to no warning (e.g., a hip fracture resulting from a fall). The caregiving role may also fluctuate as other formal and family caregivers contribute to meeting eldercare needs at different times. For conditions such as dementia, care needs typically last multiple years, well beyond the maximum duration offered by any currently available paid leave benefit.^47^ However, PSL allows for temporary absences from work that can be taken intermittently, for as little as an hour or two. Employed caregivers may need a brief work absence to deal with an unexpected medical emergency or a paid caregiver's scheduling problem; paid caregivers, who are a component of many at‐home care arrangements, exhibit a high level of turnover and scheduling volatility.^48^ The fact that employed caregivers report using sick time to deal with caregiving duties indicates the potential for a PSL mandate to encourage employees to take on, or to persist in, a period of care provision.^7^, ^41^

In other scenarios, the caregiving role may involve a care recipient experiencing one or more episodes of short‐term disability (for example, postoperative recovery and rehabilitation). PFL may be better suited to such scenarios, particularly to more predictable periods of intense caregiving, such as those associated with postacute at‐home care, or end‐of‐life care that accompanies the onset of hospice services. Both of these examples represent situations more accommodating of taking full‐time leave for a continuous period as well as the medical certification requirements that are featured in states’ PFL legislations.

Awareness of paid leave laws, specific policy provisions, and their implementation may also influence the type of leave utilized by workers. Prior literature has identified the public's lack of awareness about PFL as an important reason for low benefit uptake.^49^ In contrast, survey data from New York City showed that about 70% of workers had heard of PSL mandate passage in their city at 21 months postimplementation.^50^ Further, concerns over insufficient benefit levels, combined with a lack of job protection in some states, appear to be important barriers to PFL use.^40^ Among employees needing leave for a qualifying family or medical reason but not taking it, the fear of losing their job was the second most commonly cited reason after affordability.^43^ Finally, previous work on caregivers’ experiences with PFL in California and New Jersey indicates major challenges with the application process. Specifically, caregivers reported delays in “getting the signoff from a medical provider,” which frequently took multiple visits. In many cases, the PFL approval came only after the need for care had ended.^40^

Taken together, the differences between PFL and PSL, differences across states in program features, the diversity of caregiving scenarios, and other barriers to uptake of paid leave suggest that the effects of state‐imposed or locally imposed PSL and PFL programs on family caregiving remains an empirical question.

## Methods

### Data and Sample

Our principal data source is the HRS, a biennial, panel household survey of US adults who are aged 50 years and older when entering the sample. The HRS reinterviews respondents and their spouses every 2 years and contains extensive information on their parents’ mortality and health as well as on care provided to each parent. We used 12 waves (1998‐2020) of a version of the HRS that provides information on respondents’ state and county of residence.

Although the HRS is the best source of data to examine our research questions, certain aspects of the survey make the coding of key variables challenging. First, questions on care provision (as described in detail later) are retrospective, asking whether care was provided “since the last interview or over the last 2 years.” Because this question provides no information on the beginning or end of any caregiving episodes, or on whether such an episode is ongoing at the time of the interview, there are no point‐in‐time measures of caregiving. A positive response to the question could refer to care that was provided during all or part of as many as three different calendar years. Second, although the HRS interviews are typically fielded near the middle of an even‐numbered calendar year (the HRS “wave”), some interviews are not conducted until the next year, making it further challenging to align the timing of policy exposure with the timing of care provision using HRS waves.

To address this, we utilized the panel nature of the survey and adopt an “interval” approach to sample selection and the coding of time. Specifically, our observations refer to roughly 2‐year intervals defined by the dates of two successive interviews provided by an HRS respondent. We restricted our analysis to intervals for which the HRS respondent reports having at least one parent alive at the beginning, but not necessarily at the end, of that interval (see Supplementary Material A1 for additional details). We also limited our sample to individuals 50 years and older at the beginning of an interval and assign to each interval the county and state occupied at its beginning. The selected intervals average 23.6 months in length, and over 86% are between 18 and 30 months long; however, they range in length from 8 to 44 months.

### Paid Leave Variables

This study focuses on the effect of PFL and PSL mandates on family caregiving. We also addressed heterogeneity in the nature of paid leave exposure, reflecting whether a state's PFL law explicitly provided job protection to employees and whether respondents had access to PSL and PFL (with or without job protection) concurrently.

As shown in Tables 1, 2, 3, we considered four states that passed a PFL law during the study period. Inadequate sample sizes in the HRS ruled out consideration of Rhode Island, which passed PFL and PSL mandates in 2013 and 2018, respectively. PFL law in Washington, DC, first offered benefits in 2020, too late to appear in our HRS data. We also considered 11 states and six localities where PSL mandates were implemented during our study period. Although Connecticut passed a PSL law in 2012, it is not included as a PSL mandate state because care for a parent is not an allowable use of PSL hours under that law. A few local PSL laws are also excluded because the cities that passed them contain less than 50% of the population of the county in which they lie.

For our main analysis, we constructed two binary indicators reflecting PFL and PSL treatments, respectively. Respondent intervals associated with a nonpaid leave mandate location are coded as “untreated” in all time periods. For intervals associated with a paid leave mandate location, those ending prior to the date of a policy change are coded as “pretreatment,” whereas those that begin after the date of a policy change are coded as “posttreatment.”

Some intervals are partially treated, containing some months that precede, and others that follow, a policy change. Because of the absence of information on the timing of caregiving episodes, the degree of overlap between the caregiver outcome and the paid leave policy treatment is unknown for these intervals. In order to include these cases in the analysis, we used a midpoint approach to coding treatment status for partially treated intervals. The interval midpoint is the average value of the time covered by an interval (on the calendar‐month timeline) and therefore the best summary measure of the time period associated with the interval.

Specifically, we calculated the number of months between the midpoint of a partially treated HRS interval and the month of implementation for the policy in question (PFL or PSL). We then divided this difference by 12 and rounded the result to the nearest integer. This produces an annualized measure of relative time with respect to policy implementation, which could be negative, or zero, or positive. For both PFL and PSL treatments, partially treated intervals assigned a nonnegative value of relative time are coded as “treated.” This approach ensures that intervals in which a policy change came near the beginning had a greater chance of being coded as “treated” compared with those in which a policy change came closer to its end.

A majority of partially treated intervals were assigned a zero value of relative time and therefore were coded as “treated,” as is typical in the dynamic treatment effect literature.^51^ Some partially treated intervals were coded as “untreated” (because of their negative value of relative time) despite the fact that they included a few treated months. We retained these cases “as is” in the main analysis. We considered several alternative approaches for handling partially treated intervals in our sensitivity tests.

As shown in Tables 1, 2, 3, some states imposed both PFL and PSL mandates at various times during our study period. Also, a few localities sometimes had PSL policies in place at times that differed from those of their states. For example, in most of New York state, PFL began in 2018 and remained the only paid leave mandate in effect. New York's PFL mandate also offers job protection. However, in New York City, a PSL mandate began in April 2014, to which the PFL mandate was later added after statewide adoption. In contrast, in Westchester County, a PSL mandate was added to the existing PFL mandate in April 2019. In another instance, PFL was the only paid leave mandate in California until July 2015 (except in San Francisco, where a PSL mandate began in February 2007). The PFL mandate in California does not offer job protection. Beginning in July 2015, a PSL mandate was added to California's existing PFL mandate.

To examine heterogenous effects of different types of paid leave exposures, we created variables that distinguish among five policy regimes across different paid leave jurisdictions at different time periods: PFL without job protection (PFLNJP) and without PSL (PFLNJP only); PFL with job protection (PFLJP) but without PSL (PFLJP only), relevant only to New York state, exclusive of New York City; PSL without PFL (PSL only); and PSL in combination with PFL, with or without job protection (PSL‐PFLJP and PSL‐PFLNJP, respectively).

### Personal Care Provision

Adult children's care for parents is assessed based on the HRS respondents’ responses to questions on “whether they provided at least 100 hours of hands‐on personal care to a parent since the last interview or in the last 2 years” (that is, during the interval). The complete HRS question wording is provided in Supplementary Material A2. Personal care refers to hands‐on help with tasks such as bathing, dressing, or eating. *Provision of personal care* to a parent is a binary variable (coded “1” for affirmative responses and “0” otherwise) assessed at the end of each interval. A respondent can report positive care provision even if a parent died during the interval. We used a binary, rather than continuous, measure of care provision because a majority of caregivers are unable to recall the exact number of hours over the last 2 years and instead provide a range for the amount of care and help provided to parents.

### Covariates

We accounted for a variety of individual‐level variables potentially related to caregiving behavior. First, we included parents’ care needs. In the HRS, respondents with a parent alive (irrespective of whether they reported providing care to a parent) are asked about their parents’ health and functional status, including whether the parent needs help with basic personal needs, whether the parent can be left alone for an hour or more, and whether their cognition is impaired. These questions (described in detail in Supplementary Material A2) are asked separately for the respondent's mother and father (if alive). *Parents’ care needs* is coded as “1” when the respondent said “yes” in response to any of these questions at the beginning or the end of the interval for either mother or father, or if a parent died during the interval, and “0” otherwise. In our estimations, we also controlled for the parents’ partnered status (separately for mother and father), coded as “1” if the parent was married or partnered at the beginning of the interval and “0” otherwise.

At the respondent level, we included the respondent's age, partnership status (coded as “1” if the respondent was married or partnered at the beginning of an interval and “0” otherwise), number of living siblings, and the number of children present in the respondent's household. We also included limitations with activities of daily living (ADLs) including bathing, dressing, eating, getting in/out of bed, and walking across a room. This is measured as the total number of ADLs that the respondent reported difficulty completing; it is coded as a six‐level categorical variable that ranges from 0 to 5.

We constructed a binary variable approximating an individual's eligibility for FMLA leave using responses to HRS questions about tenure at one's current job, hours worked per week, annual number of weeks worked, and the size of the employer's firm. Nonemployed and self‐employed individuals are coded as ineligible for the FMLA. Unfortunately, we were unable to assign FMLA eligibility for 15% of those potentially eligible because of item missingness (mainly on firm size). Given both measurement error and the potential bias arising from the large number of missing observations on FMLA eligibility, we controlled for this variable in only some specifications.

We also controlled for state‐level variables including state adoption of the Personal Care Services (PCS) Medicaid option (which provides an offer of formal care for hands‐on assistance with ADLs or instrumental ADLs), Affordable Care Act (ACA) Medicaid expansion status, minimum wage levels, unemployment levels, and poverty rates. The PCS Medicaid option and Medicaid ACA expansion are included because they have the potential to influence family caregiving either directly or through their influence on the availability and accessibility of formal care.^52^, ^53^, ^54^, ^55^ Beyond PCS, Medicaid 1915(c) waivers are the main instruments through which states offer home and community‐based services under their Medicaid program. We did not control for the presence of these waivers given considerable variation in the number and type of waivers across states. In contrast, the PCS Medicaid option is a state plan benefit; that is, unlike a waiver, states can neither target specific populations or regions nor impose limits on program enrollment. Further, every state administered at least two 1915(c) waivers by 1999 (1 year after our study period began), with 45 offering personal care through waivers, thus leading to little variation in waiver adoption status across states for the analysis conducted in our study.^56^, ^57^ Other than parent care needs, all other covariates are assessed at the beginning of the interval; for additional details on covariates (including data sources), see Supplementary Material A2.

### Empirical Strategy

To estimate the causal effect of state‐level PFL and PSL mandates on personal care provision to older parents, we exploited the variation in the timing of mandate implementation for each policy using a difference‐in‐differences (DD) design. This design compares changes in the probability of parent care between locations with and without these policies, before versus after policy implementation. Because we had multiple states and localities that begin treatment (implementation of a PFL or PSL mandate) at different times, we applied a generalized DD design (also known as the staggered‐adoption DD design), which is analyzed using a fixed‐effects regression.^58^ Specifically, our main DD estimation uses the following regression equation:

(1) $$Y_{ist}=\beta_{0}+\beta_{1}PFL_{ist}+\beta_{2}PSL_{ist}\;+\;\beta_{3}X_{ist}+\beta_{4}Z_{ist}+\alpha_{i}+\lambda_{t}\;+\theta_{s}+\;\varepsilon_{ist}.$$

In equation (1), *Y_ist_* is a binary indicator of personal care provision to parents for individual *i* living in location *s* at time period *t*. *PFL_ist_* and *PSL_ist_* reflect an individual's status with respect to the PFL or PSL treatment in location *s* at time *t*, thus providing the treatment effect of these policies on personal care provision. Analogous to the canonical 2 × 2 DD setup, these variables are the product of time‐invariant group (location) dummies and group invariant time period dummies. *X* is a vector of time‐varying respondent‐ and parent‐level control variables, whereas *Z* is a vector capturing time‐varying economic conditions and state policies on long‐term care.

We also included individual (α), location (θ), and time (δ) fixed effects. Individual fixed effects account for otherwise unmeasured propensities for care provision and for residing in places with certain policy environments. Location‐specific fixed effects capture time‐invariant differences across states and localities. Although the PFL laws considered are all state‐level policies, the PSL laws include some state‐level and some county‐level policies. Therefore, instead of state fixed effects, we used location‐based fixed effects. For states containing one or more counties that passed a PSL law (e.g., Pennsylvania), the state indicator is recoded to reflect the distinction between counties in which a local PSL law was passed (i.e., Philadelphia) and the untreated remainder of the state (e.g., all Pennsylvania counties other than Philadelphia.). Both individual and location fixed effects can be included because some individuals move over time. Year fixed effects (based on the calendar year associated with the respondent‐interval midpoint) capture common trends or secular patterns shared throughout the population. We estimated linear probability models, which have been empirically shown to provide reliable estimates of marginal effects in a wide range of circumstances, to estimate all models.^59^ Robust standard errors are clustered at the state level.

We estimated three main models. We first examined the effect of PSL and PFL while controlling for only individual, location, and year fixed effects. Second, we included time‐varying covariates in addition to the fixed effects. Finally, although a person must be employed in order to take any form of leave from work, employment status can change within a 2‐year period such as that used to define our time intervals. Moreover, sampling based on a point‐in‐time measure of employment status risks the introduction of selection bias to our estimates. As a robustness test, we paired our second model with model 3, for which we restricted the sample to individuals who reported working for pay at the beginning of the interval. The pooled sample size for our main estimation (model 2) comprises 38,183 observations.

A causal interpretation of DD estimates relies on a parallel trends assumption, namely that outcomes in the treatment and control states would have evolved similarly if PFL and PSL policies had not been implemented. Although neither necessary nor sufficient for claiming causality, demonstrating the existence of parallel pretreatment trends strengthens those claims.^60^ We used an event‐study approach to decompose average effects over the entire study period into effects for each year before and after policy implementation. We applied the Sun and Abraham interaction‐weighted estimator to account for staggered treatment timing and heterogeneous effects across locations.^61^ Using this method, we estimated separate event studies for PFL and PSL treatments. In the Sun and Abraham approach, treated locations are categorized into cohorts based on their initial treatment timing.^61^ A cohort‐specific average treatment effect on the treated, defined in relative treatment time terms and comparing a treatment unit's potential outcome at a point in time if it received treatment in a particular time period with the counterfactual outcome if the unit never receives treatment, is estimated. This estimate removes contamination from spillover effects from earlier time periods and produces estimates that are unbiased and consistent estimators of the average treatment effect on the treated for each cohort.

An event‐study approach necessitates that every interval be assigned a relative time (i.e., leads and lags indicating, for each location interval observation, the number of time periods by which it precedes or follows the policy change).^51^ In order to code relative treatment times for each respondent interval, we required a time index associated with each interval. We utilized the midpoint approach described earlier in coding treatment status for partially treated intervals. In this case, for all intervals (not just partially treated ones), we calculated an annualized measure by computing the number of months between the midpoint of an interval and the implementation time of a policy, dividing the difference by 12, and rounding to the nearest integer. This produces a number (which can be negative, or zero, or positive) that we used to code the number of years prepolicy (or postpolicy) implementation. This approach for coding relative time for event‐study analysis is consistent with our main approach for assigning treatment status and thus our main DD regressions. Note, relative treatment times for respondent intervals associated with untreated locations (locations with no paid leave policies) are coded as zero throughout.

We also tested for two types of heterogeneous impacts. The first, heterogeneity of paid leave policies, uses the five combinations of PFL (with or without job protection) and PSL defined earlier. For the second, we investigated whether selected population subgroups exhibit heterogeneous responses to the overall PFL and PSL treatments. These dimensions included the child's gender, age, partnership status, and education (completed college vs. did not complete college).

Finally, we conducted four sets of sensitivity checks. First, we removed HRS respondents who had ever moved and reestimated our main DD model with only individual and year fixed effects. This approach addresses the potential for endogenous move behavior not captured by individual fixed effects in the main model. Second, instead of including both PFL and the PSL treatment variables in the same regression, we estimated each treatment effect via two separate regressions. Third, we tested whether the results are sensitive to our coding of treatment status for partially treated intervals using three alternative approaches. In the initial approach, we considered all partially treated intervals—even those that ended only a month or two after the policy change—as “treated.” Because this approach considers intervals that had as little as 1 month of policy exposure as “treated,” we expected our estimate of a behavioral response as a result of PFL or PSL policy change to be attenuated. Next, we discarded all partially treated intervals associated with a negative value of relative time. For our main results to be robust, dropping these problematic intervals should have minimal effect on our findings. Finally, instead of assigning a binary treatment status to partially treated intervals using the midpoint approach, we assigned each partially treated interval (for both PFL and PSL) a fractional treatment status given by the number of treated months (numerator) as a proportion of the total number of months in the interval (denominator). In the fourth sensitivity check, because COVID‐19 could impact caregiving and labor market behavior of family caregivers, we reestimated our main model after dropping intervals that end with the 2020 HRS interview information.

## Results

Table 4 provides summary statistics for all covariates for the full sample and separately for ever‐treated PFL and PSL areas. In the full sample, the average age of respondents was approximately 58 years old and about 73% were partnered. Over half the respondents reported having a parent with care needs. Approximately 64% of respondents reported working for pay at the beginning of an interval. About 13% of respondents reported providing care to any parent, with 11% providing care to their mother, 3% provision care to their father, and 1% providing care to both parents. With regard to the presence of other potential caregivers, respondents reported an average of two living siblings. Overall, respondents in PFL and PSL mandate areas had similar characteristics compared with those in the full sample, although mandate areas were more likely to have adopted other state‐level policies such as the ACA and PCS.

**Table 4:** Summary Statistics

Characteristic	Full Sample	PSL Areas	PFL Areas
Parent‐level variables			
Parent care need, %	53	52	54
Mother's partnered status, %	22	23	23
Father's partnered status, %	24	24	24
Respondent‐level variables			
Age, mean (SD)	58.43 (5.63)	58.29 (5.54)	58.10 (5.40)
Number of living brothers, mean (SD)	1.60 (1.48)	1.62 (1.47)	1.67 (1.51)
Number of living sisters, mean (SD)	1.67 (1.56)	1.69 (1.56)	1.72 (1.60)
Number of children in HH, mean (SD)	2.94 (1.94)	2.89 (1.96)	2.87 (1.96)
Partnered status, %	73	72	72
Female, %	58	59	58
Less than high school education, %	14	14	15
College and above education, %	27	28	29
Non‐Hispanic Black, %	18	17	14
Other race, %	4	5	6
Worked for pay at baseline, %	64	64	64
ADL limitations, % reporting difficulty			
0	90	90	89
1	5	5	6
2	2	2	3
3	1	1	1
4	1	1	1
5	0.00	0.00	0.00
Personal care provision to *any* parent, %	13	13	13
Personal care provision to mother, %	11	11	11
Personal care provision to father, %	3	3	3
Personal care provision to *both* parents, %	1	1	1
State‐level variables			
Minimum wage	6.66	7.38	7.64
Unemployment rate	5.89	6.55	6.75
Poverty rate	13.30	13.14	13.47
ACA expansion, %	15	28	30
Medicaid personal care state plan, %	75	93	100
*N*	42,867	17,587	10,380

ACA, Affordable Care Act; ADL, activity of daily living; HH, household; PFL, Paid Family Leave policy; PSL, Paid Sick Leave policy; SD, standard deviation.

Personal care provision reflects the provision of at least 100 hours of hands‐on personal care to a parent since the last interview.

Table 5 reports the results for our three main models. Model 1, which omits covariates, indicates that PSL mandates produced a statistically significant increase in care provision to older parents of about four percentage points. Among respondents in PSL mandate areas, approximately 13% provided personal care to parents before the implementation of the law. Thus, a PSL mandate was associated with a relative increase of approximately 32% in personal care provision. In contrast, PFL mandates had little impact on care provision to parents for the full sample. Specifically, the PFL estimate was small, negative, and statistically insignificant. In model 2, which includes all covariates (except FMLA eligibility), the PSL coefficient is slightly larger (4.7 percentage points, a 35% relative increase), whereas the PFL coefficient continues to remain negative and imprecise. When FMLA eligibility is included in the estimation (*N* = 34,818; Supplementary Material A3), the coefficient for PSL mandate drops slightly to 0.046 (*p* = 0.004). The coefficient for PFL mandate increases to 0.014 but remains statistically insignificant (*p* = 0.302).

**Table 5:** Changes in Probability of Personal Care Provision to Parents Associated With PFL and PSL Mandates

Treatment Effect	Model 1: Full Sample, No Covariates	Model 2: Full Sample, With Covariates	Model 3: Baseline Workers, With Covariates
PFL mandate (SE)	−0.002 (0.018)	−0.001 (0.013)	−0.014 (0.027)
Pretreatment mean, %	13	13	12
PSL mandate (SE)	0.041^**^ (0.014)	0.047^**^ (0.014)	0.053^**^ (0.019)
Pretreatment mean, %	13	13	11
*N*	40,077	38,183	23,338

PFL, Paid Family Leave policy; PSL, Paid Sick Leave policy; SE, standard error.

^*^ *p* < 0.05, ^**^ *p* < 0.01, ^***^ *p* < 0.001.

All estimations are restricted to respondents aged 50 years and older and to those with at least one parent alive at baseline. Covariates in models 2 and 3 include the following: parent care need, mother's partnered status, father's partnered status, respondent age, number of brothers, number of sisters, number of children, respondent activities of daily living, minimum wage, unemployment rate, poverty rate, Medicaid Affordable Care Act expansion status, and the presence of Medicaid Personal Care Services state plan option. All regressions control for individual, location, and year fixed effects. “Pretreatment mean” refers to the mean of personal care provision in PFL‐ and PSL‐treated areas before policy introduction. Standard errors (in parentheses) are clustered at state level.

Model 3 of Table 5 shows results for respondents employed at baseline. Because paid leave policies (whether PFL or PSL) are provided as an employment benefit, we expected our results to be strongest among this subgroup of individuals. As expected, we found that a PSL mandate increases the probability of personal care provision to parents by approximately 5.3 percentage points, reflecting a relative increase in personal caregiving of almost 40%. Despite being based on a smaller sample size, this estimate remains statistically significant. The PFL coefficient is also larger; however, it remains statistically insignificant. Among the sample of baseline workers, the inclusion of FMLA eligibility does not change the coefficient or statistical significance for PSL treatment (*β* = 0.053, *p* = 0.013, *N* = 20,074). The coefficient on PFL treatment changes to 0.01 but remains statistically insignificant (*p* = 0.796). The coefficient for FMLA eligibility is not statistically significant in any estimation. Full regression results, including estimations with FMLA eligibility as an additional control variable, are provided in Supplementary Material A3.

**Figure 1:**
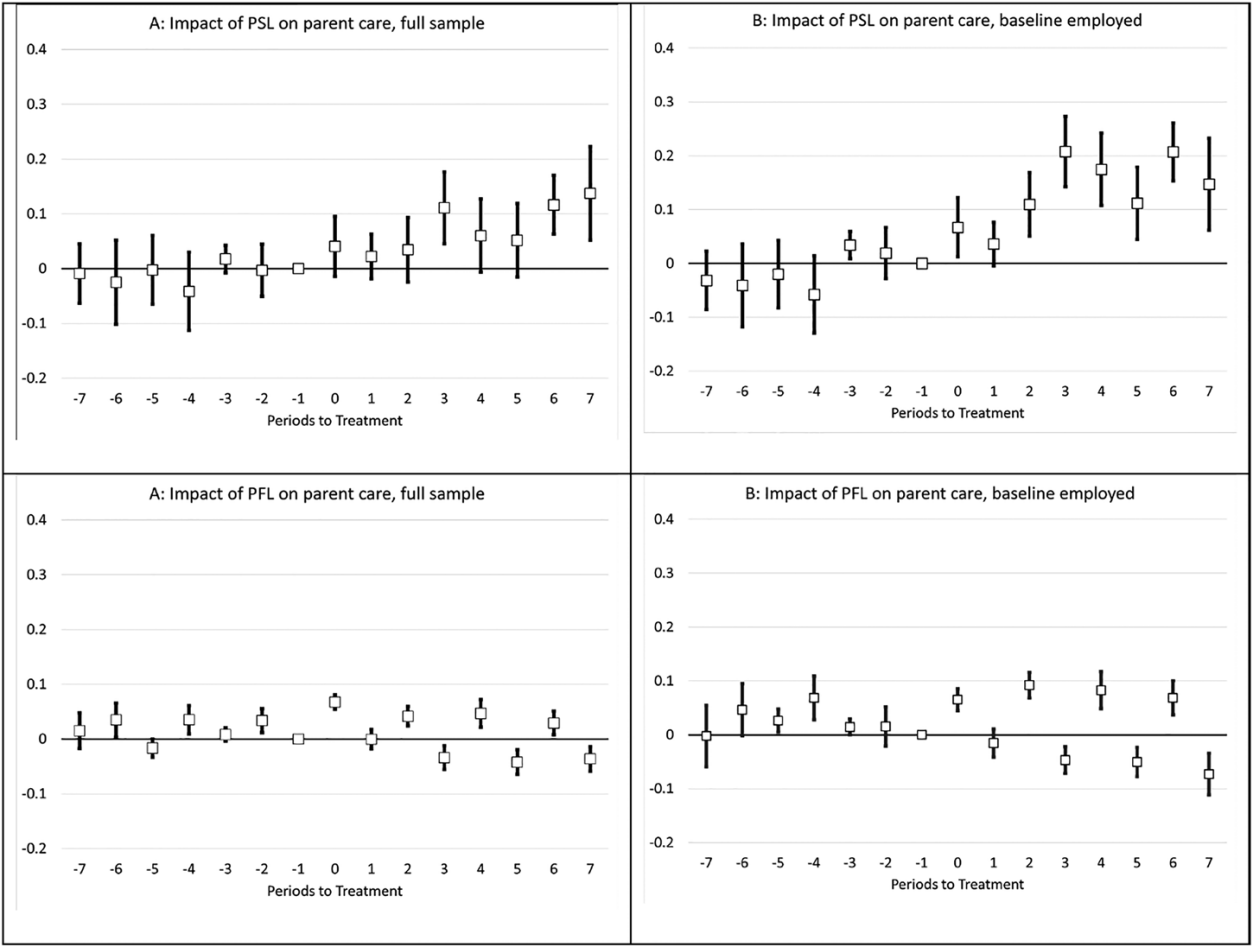
Event‐Study Estimates of Adjusted Yearly Changes in the Probability of Personal Care Provision Associated With PSL Adoption (Upper Panel) and PFL Adoption (Lower Panel) for (A) Full Sample and (B) for Those Employed at Baseline Estimates are from event studies performed by authors using the methods of Sun and Abraham.^62^ For each location, the year immediately before the adoption of the PSL mandate is used as the reference year (denoted as “−1”). Each square represents the estimated change in the probability of personal care provision to parents in areas with PSL (PFL) mandates relative to areas without them in the years pre‐ and postimplementation relative to the reference year. The estimates are adjusted for PSL (PFL) adoption, parent care need, mother's partnered status, father's partnered status, respondent age, number of brothers, number of sisters, number of children, respondent activities of daily living, minimum wage, unemployment rate, poverty rate, Medicaid Affordable Care Act expansion status, and the presence of Medicaid Personal Care Services state plan option. All regressions control for individual, location, and year fixed effects. The lines around each estimate represent 95% confidence intervals. Robust standard errors are clustered at the state level. PFL, Paid Family Leave policy; PSL, Paid Sick Leave policy.

Figure 1 shows plots of event‐study coefficients, illustrating dynamic treatment effects of PSL and PFL treatments, respectively, for up to 7 years before and after implementation. In this analysis, all estimates reflect changes relative to a reference year, which is the year before implementation (the “−1” period). The upper half of Figure 1 pertains to PSL effects, whereas the lower half pertains to PFL effects; in both cases, panel A represents the full sample and panel B the smaller sample of those employed at baseline.

Starting with PSL (the upper left graph), we saw that the probability of care provision increased immediately 1 year after PSL passage (though the estimate is not statistically significant), with larger increases about 3 years postimplementation. A similar pattern of results is seen for baseline workers, with much larger coefficients in the postperiod compared with the full sample, consistent with the overall DD result. The event‐study estimates also allow us to test for parallel pretreatment trends in the parent care outcomes. We found no evidence of differential trends in personal care provision between PSL and comparison areas before the implementation of the mandate, lending support to our claim that the estimated PSL effects are causal.

In contrast with PSL, the PFL event‐study results show no consistent pattern in the postperiod for either the full sample or the subsample of initially employed workers despite the presence of several statistically significant coefficients with tight confidence intervals. The unusual pattern of event‐study results for PFL is almost entirely because of compositional issues: the estimates for even‐numbered posttreatment periods—generally suggesting a positive response to PFL—are almost exclusively for New York (periods 0 and 2) or New Jersey (periods 4 and 6) residents, whereas the estimates for odd‐numbered periods—suggestive of a negative response to PFL—are almost exclusively for California residents. These features of our data are, in turn, a reflection of the timing of HRS interviews and the timing of policy changes in the respective states. We did not observe such a pattern for PSL because several states passed PSL laws in both odd and even years.

Next, we examined the potential for heterogeneous treatment effects based on different types of policy exposure. Table 6, column 1, provides results for disaggregated PFL and PSL variables for the full sample. We found that coefficients for PFLNJP only and PFLJP only are small and statistically insignificant. PSL only leads to an increase in personal care provision by approximately three percentage points, though the coefficient is statistically significant only at 10%. However, both PSL‐PFLJP and PSL‐PFLNJP are associated with large statistically significant increases in personal care provision. Specifically, PSL‐PFLJP and PSL‐PFLNJP are associated with an increase in personal care provision to parents by about six and five percentage points, respectively. When we examined the subsample of respondents employed at baseline (Table 6, column 2), we found that an increase in care provision is mainly attributable to PFLJP only (which, in this case, refers to upstate New York), PSL‐PFLJP, and PSL‐PFLNJP treatments. Coefficients for PSL only and PFLNJP only are small and statistically insignificant. The pattern of results remains similar when FMLA eligibility is included in these estimations as a control variable, except PFLJP only is now associated with statistically significant increase in personal care provision in both the full and baseline worker samples (see Supplementary Appendix 3). The differences between the PFLJP and PFLNJP results provide a partial explanation for the odd pattern of posttreatment coefficients found in the event‐study graphs found in Figure 1: positive coefficients are associated mainly with PFLJP states (New York), and negative coefficients are associated mainly with PFLNJP states (California, New Jersey, and Washington).

**Table 6:** Changes in Probability of Personal Care Provision to Parents Associated With Different Types of Paid Leave Exposures

Treatment Effect	Model 1: Full Sample	Model 2: Baseline Workers
PFL not job protected only (SE)	−0.009 (0.008)	−0.035 (0.018)
Pretreatment mean, %	14	12
PFL job protected only (SE)	0.014 (0.012)	0.036^*^ (0.016)
Pretreatment mean, %	13	13
PSL only (SE)	0.0335 (0.019)	0.0355 (0.027)
Pretreatment mean, %	13	12
PSL‐PFL job protected (SE)	0.064^***^ (0.017)	0.068^**^ (0.021)
Pretreatment mean, %	13	12
PSL‐PFL not job protected (SE)	0.052^**^ (0.015)	0.038^*^ (0.019)
Pretreatment mean, %	12	10
*N*	38,183	23,338

PFL, Paid Family Leave policy; PSL, Paid Sick Leave policy; SE, standard error.

^*^ *p* < 0.05, ^**^ *p* < 0.01, ^***^ *p* < 0.001.

Estimations are restricted to respondents aged 50 years and older and to those with at least one parent alive at baseline. Covariates in models 1 and 2 include the following: parent care need, mother's partnered status, father's partnered status, respondent age, number of brothers, number of sisters, number of children, respondent activities of daily living, minimum wage, unemployment rate, poverty rate, Medicaid Affordable Care Act expansion status, and the presence of Medicaid Personal Care Services state plan option. All regressions control for individual, location, and year fixed effects. “Pretreatment mean” refers to the mean of personal care provision in treated areas before introduction of policy regime. Standard errors (in parentheses) are clustered at state level.

Heterogeneity of responses by respondent subgroups is addressed in Table 7. Our main result on the effect of PSL passage on personal care provision to parents is largely attributable to women and those younger than 65 years of age. There is no detectable association between PSL passage and personal care provision to parents among men or for those 65 years and older. Further, although PSL passage is associated with an increase in the probability of personal care provision among both partnered and unpartnered respondents, the treatment effect is larger for unpartnered respondents. PSL passage also has a larger effect on personal care provision among those who attained college education or higher (relative to those who did not complete college). There is no detectable association between PFL passage and personal care provision among any of these respondent subgroups.

**Table 7:** Difference‐in‐Differences Estimates of Changes in Probability of Personal Care Provision to Parents Associated With PFL and PSL Mandates by Subgroups

Treatment Effect	Model 1: Women	Model 2: Men	Model 3: <65 yrs	Model 4: ≥65 yrs	Model 5: Partnered	Model 6: Unpartnered	Model 7: College	Model 8: No College
PFL mandate (SE)	−0.011 (0.011)	0.011 (0.025)	0.012 (0.010)	−0.006 (0.020)	−0.0134 (0.010)	−0.003 (0.043)	−0.019 (0.037)	0.005 (0.010)
Pretreatment mean, %	16	9	12	17	12	16	12	13
PSL mandate (SE)	0.063^*^ (0.029)	0.027 (0.020)	0.057^***^ (0.016)	0.003 (0.040)	0.045^*^ (0.020)	0.055^**^ (0.017)	0.075^**^ (0.028)	0.033^*^ (0.015)
Pretreatment mean, %	16	8	11	17	12	15	13	13
*N*	22,492	15,691	31,909	5,154	27,700	9,764	10,426	27,756

PFL, Paid Family Leave policy; PSL, Paid Sick Leave policy; SE, standard error; yr, year.

^*^ *p* < 0.05, ^**^ *p* < 0.01, ^***^ *p* < 0.001.

Estimations are restricted to respondents aged 50 yrs and older and to those with at least one parent alive at baseline. Covariates in all models include parent care need, mother's partnered status, father's partnered status, respondent age, number of brothers, number of sisters, number of children, respondent activities of daily living, minimum wage, unemployment rate, poverty rate, Medicaid Affordable Care Act expansion status, and the presence of Medicaid Personal Care Services state plan option. All regressions control for individual, location, and yr fixed effects. “Pretreatment mean” refers to the mean of personal care provision in PFL‐ and PSL‐treated areas before policy introduction. Standard errors (in parentheses) are clustered at state level.

Results from sensitivity tests are provided in the Supplementary Material, Table A4. Removing respondents who moved between states did not substantially change our main results (column 1). We also obtained similar results when we assessed the effect of PFL and PSL treatments using two separate regressions (columns 2 and 3). Under all three alternative approaches to dealing with partially treated intervals (columns 4–6), our main results are preserved. Finally, dropping intervals that end with the 2020 HRS wave produces results similar to our main results, albeit with slightly stronger treatment effects (column 7).

## Discussion

Our findings indicate that the prevalence of parental caregiving increased in states and localities that adopted laws requiring employers to provide paid sick time during the last two decades. Averaging over all locations and treated periods, the increase ranges from four to five percentage points; however, because the pretreatment prevalence of parental caregiving is about 13%, the policy impact represents a relative increase of at least 32%. This is reasonable in light of results from other studies that show a 30% increase in sick leave coverage rates within the first 2 years of PSL adoption, consequently leading newly covered employees to take as many as two additional sick days per year. Moreover, our event‐study results indicate that the PSL impacts are unlikely to be an artifact of preexisting but unmeasured differences between treated and untreated locations. The PSL impact is dominated by states during periods when PSL and PFL programs are concurrently offered.

Results from our main model also indicate that states’ PFL mandates did not produce detectable changes in parental caregiving behavior on average (indeed, estimated policy effects are small and statistically insignificant). However, these findings should not be interpreted to mean that PFL laws are ineffective. Our main result is largely attributable to respondents in states and time periods representing exposure to only a non–job‐protected PFL law. In contrast, the implementation of job‐protected PFL—both by itself (upstate New York) and in combination with PSL (New York City)—is associated with an increase in the probability of personal care provision to parents. This finding is consistent with previous studies that highlight the importance of job protection for workers considering family leave.^40^, ^43^, ^62^

We also observed an increase in care provision for respondents in states with preexisting PFL laws (albeit not job protected) but only in periods when the state concurrently implemented a PSL law. These results are heavily influenced by California given its large size and relatively early PSL adoption. Specifically, California was one of the first states (after Washington, DC) to pass PSL in 2015, thus lending the majority of treated observations in the post‐PSL and, consequently, post–PSL‐PFLNJP period. The event‐study results for PSL passage show that the effect grows over time, underscoring the importance of a large number of posttreatment observations. As a result, the positive association between care provision and the combined—PSL and non–job‐protected PFL—treatment likely reflects the effect of PSL passage in California. It is also possible that this finding is informed by changes made by the state to its PFL law after 2015. For instance, over 2014–2016, California extended a major outreach effort to increase PFL awareness and inform residents about the availability of family leave benefits. California also increased the PFL wage‐replacement rate from 55% to approximately 60%‐70%, depending on income, and extended the maximum leave from 6 to 8 weeks. However, changes to California's PFL wage replacement and leave duration went into effect quite late with respect to our study period (January 2018 and July 2020, respectively).^63^ Disentangling these effects is beyond the scope of this current study. Future research should assess how specific policy features influence care outcomes.

The lack of association between parent care provision and states with only non–job‐protected PFL mandates appears to be inconsistent with previous work showing a decline in aggregate nursing home use among older adults following California's PFL law in the period when a PSL mandate was yet to be passed in the state.^35^ It is possible that parent care efforts are dispersed across siblings, such that each sibling's PFL‐induced care response is too small to be detected, whereas collectively, these efforts produce a significant reduction in the parent's chances of nursing home occupancy. In addition, it is also possible that adult children use paid time off to vet, hire, and train formal caregivers in order to complement their own care effort. In that scenario, although the probability of family caregiving would remain unchanged, the overall increase in the use of community care may contribute to a reduction in future nursing home use.^64^ Further, the present study only focuses on individuals 50 years and older. Younger caregivers (who comprise almost half of all family caregivers) may be more responsive to paid leave programs.^65^

Consistent with our main results, we did not find any detectable association between PFL passage and personal care provision to parents among any analyzed respondent subgroups. The increase in personal care provision associated with PSL mandates was largest among women. This is not surprising considering female caregivers have nearly three times the odds of reporting absenteeism relative to male caregivers.^5^ Evidence from recent work also indicates that PSL mandates significantly benefited women by narrowing the gender gap in sick leave coverage in adopting states and localities.^34^

We also found that the effect of PSL passage on personal care provision is more pronounced among younger adult children, potentially relating to the fact that younger respondents are more likely to be working and thus more likely to be exposed to paid leave policies. Additionally, despite a smaller sample size, the PSL treatment effect was larger among unpartnered individuals. This finding adds to the robustness of our overall results, as we would expect those with no other potential wage earner in the family to be more likely to utilize paid leave policies. This is particularly relevant in the context of previous work indicating that parent's health status leads to negative financial consequences among unmarried adult children.^66^

Our results show the effect of PSL passage on personal care provision are stronger among those completing college education or more (relative to those who do not have a college degree). This is surprising, as less‐educated individuals are more likely to work in industries with low access to PSL without a policy intervention (e.g., accommodation and food services industries) and are thus more likely to gain coverage after the passage of a state‐ or municipalitywide PSL law, relative to those with higher education.^67^, ^68^ At the same time, higher education is a predictor of better health, and therefore, it is possible that less‐educated workers utilize paid leave to address their own health needs, whereas more‐educated workers use paid time off to support their parents and other family members.^69^

Our study has multiple limitations. The combination of the HRS sample size, panel coverage (1998‐2020), and the timing of policy changes influences our findings. Of the 21 distinct policy changes included in our analysis, over half can only be detected in the last two of our 12 HRS surveys, and over three‐quarters occur in time for the last three surveys. The HRS sample is large enough for us to test for differences between PFL with and without job protection but not for differences by programmatic features such as wage‐replacement rates, PSL accrual rates, or exemptions according to firm size. Additional waves of data collection will allow for more thorough investigations of program impacts as well as their posttreatment trajectories. Inadequate sample sizes also ruled out consideration of states like Rhode Island, which passed both PSL and PFL mandates during our study period. We also did not address other types of caregiving, such as that delivered by spouses. However, spousal caregiving is more common at older ages, when individuals are less likely to be working. Another limitation is that the HRS question on caregiving does not provide information on the timing of caregiving episodes or on whether caregiving episodes remain in progress at the time of the interview. We also did not have detailed information on the intensity of care provided by family caregivers or any measures of the duration of parents’ care needs. Additionally, we did not observe sample members’ actual leave‐taking behavior, whether through use of either sick days or paid family leave entitlements, for purposes of providing parent care; instead, we observed only the care outcomes, leaving implicit the connection between paid leave mandates and care behavior. We believed that these limiting features of the HRS data are more than outweighed by its strengths, including its lengthy panel period and its measures of caregiving, parents’ care needs, and respondents’ locations. Finally, our analysis does not account for several other state policies that may influence family caregiving directly or indirectly.

## Conclusion

These limitations notwithstanding, our findings have implications for individuals and their families and for state policymakers considering improved policy protections for family caregivers.^11^ Increased caregiving among employed children of elderly parents implies a reduction in the stress of balancing competing responsibilities. Given well‐documented adverse consequences of chronic stress for both psychological and physical health, our findings imply a range of beneficial health outcomes for caregivers.^70^ Investments in paid leave can also influence the well‐being of older adults by contributing to the long‐term services and supporting environmental context.^71^ The design and implementation of these policies should be carefully considered. Despite PSL's flexible design with full wage replacement and built‐in job protection, it is unlikely to support longer‐term caregiving. In contrast, PFL, which offers more time off, may not be effective by itself without features like job protection. Overall, when considered in the context of only modest increases in employer costs, well formulated and executed paid leave policies may play an important role in improving a variety of health outcomes.^30^, ^32^

## Conflict of Interest Disclosures

The authors declare no conflicts of interest.

## Supporting information

Supplementary Information
