# Supplementary material for: Paid Leave Mandates and Care for Older Parents
Source: Milbank Q. 2024 Jun 20;102(3):732–64. doi: 10.1111/1468-0009.12708 (PMC11576588; doi:10.1111/1468-0009.12708)
Supplement: Supplementary file 1 — Supplementary Information [file MILQ-102-732-s001.docx]

**Supplementary Materials**

Supplementary Material A1: Interval coding of HRS data; Coding of Treatment, Pre- and Post-treatment periods, and Partially Treated Intervals

*Interval Coding*

HRS is a biennial, panel survey of US adults who are aged 50 and older when entering the sample. HRS surveys (“waves”) are typically fielded in even years (e.g., 1998, 2000, 2002 and so on). In Figure A1.1, a timeline is shown, divided into one-month intervals. Generic HRS interviews for waves “W” and “W + 2” are shown, spanning a 23-month period. The red stripe represents this HRS “interval.” Both the wave W and wave W + 2 interviews occur in the middle of a calendar year, as is typical for HRS interviews (depicted as months 6 and 29 on the timeline shown). Calendar years “Y”, “Y + 1”, and “Y + 2” are also shown, represented by the blue strips. The first month of each calendar year is, of course, January; in the diagram, month 1 is January of year “Y”, and so on.

For our analysis, the challenge in employing the HRS is that key questions on care and help provision are retrospective in nature and span the time “since the last interview” or over the “last two years” (see Supplementary Material A2). As a result, the response to these questions could contain an informal care activity that was done at any time over all or parts of 3 different calendar years. For example, an individual interviewed in June 2018 for the 2018 HRS wave could be responding about a care activity that took place in 2018, 2017, or 2016. In this scenario, if a paid leave law was introduced in a state in 2017, then coding responses from the subsequent wave (in this case, HRS 2018 wave) as the start of policy exposure may not accurately line up with a care report potentially referring to an activity conducted in 2016. We might inadvertently code a positive report of personal care provision to coincide with policy introduction when that may not have been the case. This makes it difficult to align the timing of the outcome with the timing of policy implementation (which is based on calendar year).

The HRS caregiving question does not provide information on the timing of episodes of caregiving, nor on whether caregiving episodes remain in-progress at the time of the interview. Therefore there is no point-in-time measure of caregiving. Moreover, in addition to the two-year look-back window, the variability in duration since last interview (some look-back periods – “time since last interview” – span as many as 44 months), the timing of the interview (while a majority of HRS interviews typically occur in the middle of a calendar year, some occur in subsequent years), the variation in year/month of policy changes (e.g., even vs. odd years, January vs June passage) make it further challenging to code treatment status based solely on HRS waves.

Thus, we use the panel nature of the HRS and divide time into intervals, which is defined by data provided by a respondent in two successive interviews (“W” and “W+2”). For each interval-observation, the beginning year and moth is provided by the last interview, and the ending year and month is provided by the current interview. Interview data collected during the “W” interview produces several of our beginning-of-interval variables, while interview data collected during the “W + 2” interview provides the information needed to identify care activity (accounting for the retrospective nature of the caregiving question).

*Figure A1.1: Illustration of interval coding of HRS data*


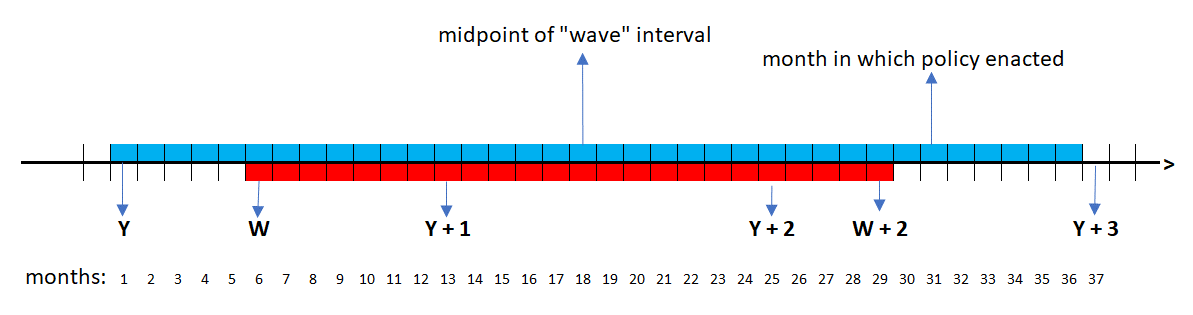


*Coding of “Treatment”*

For intervals associated with paid level mandate location, those that end before the date of policy implementation are considered pre-treatment, and those that begin after the date of policy implementation are considered post-treatment. Figure A1.1 also illustrates how this works. The implementation of a paid leave policy, in this case in month 3 of the interval that begins with wave “W + 2.” For this policy, the “W” interval is, therefore, a pre-treatment interval.

Some intervals are partially treated, i.e., they contain the policy event. This leads to ambiguity as to how to code these intervals, and we provide various approaches (one preferred approach and multiple sensitivity checks) to address this. Our preferred approach (described below) employs the interval midpoint to assign treatment status to partially treated intervals. Respondent intervals associated with a non-paid leave mandate location are coded as “untreated” in all time periods.

Specifically, we calculated the number of months between the midpoint of a partially treated interval and the implementation time of the policy in question (PFL or PSL). We then divided this difference by 12 and rounded the result to the nearest integer. This produced an annualized measure of relative time with respect to policy implementation, which could be negative, or zero, or positive. For both the PFL and PSL treatments, partially intervals assigned a nonnegative value of relative time were coded as “treated.”

We chose the midpoint because it is the same as the average value of the time covered by an interval (on the calendar-month timeline), and therefore the best summary measure of the time period associated with the interval. The use of this approach implies that partially treated intervals with a large amount of exposure to a paid leave policy (i.e., where the policy change came at the beginning of the interval) had greater chance of being coded as “treated” relative to those with little exposure to a paid leave policy (i.e., where the policy change came closer to the end). Further, as described below, using the midpoint to code treatment status for partially treated intervals also allows for consistency between our DD and event study estimations.

As expected, a majority of partially treated intervals were assigned a zero value of relative time and therefore were coded as “treated,” following the practice followed in the dynamic treatment-effect literature (Clarke & Tapia-Schythe, 2021). Some partially treated intervals were coded as “untreated” (due to their negative value of relative time) despite the fact that they included a few treated months. We considered several alternative approaches for handling partially treated intervals in our sensitivity tests (including, coding all partially treated intervals as treated, dropping partially treated intervals associated with a negative value of relative time, and a proportional approach – where treatment assignment for partially treated intervals is not dependent on the interval midpoint).

*Pre- and Post-Treatment Periods*

We also use the midpoint approach to code relative time for our event study estimation. A causal interpretation of DD estimates relies on the parallel trends assumption: that outcomes in the treatment and control states would have evolved similarly if PFL and PSL policies had not been implemented. While neither necessary nor sufficient for claiming causality, the existence of parallel pre-treatment trends strengthen those claims. We used an event-study approach to decompose average effects over the entire study period into effects for each year before and after policy implementation. An event study approach necessitates that every interval be assigned a relative time (i.e., leads and lags indicating that a given location – associated with an interval – was a given period away from the start of policy passage in a respective time period) (Clarke & Tapia-Schythe, 2021).

In order to code relative treatment times for each respondent-interval, we require a time index associated with each interval. As discussed above, the midpoint is the best summary measure of the time period associated with the interval. For all intervals, we calculate an annualized measure generated by comparing the midpoint of each respondent interval and the timing of policy passage (i.e., the number of months between the midpoint of an interval and the implementation time of the policy, which is then divided by 12 and rounded to the nearest integer). We are left with a number (which can be negative, or zero, or positive) that we use to code the number of years pre- (or post-) policy implementation.

This approach for coding relative time (for event study analysis) is consistent with our main approach for assigning treatment status, and thus our main DD regressions. When a state’s policy change occurred after an interval’s end date (i.e., pre-treatment intervals), then the interval’s relative time measure is necessarily negative, and when the policy change preceded an interval’s beginning date (post-treatment intervals), that interval’s relative time measure is necessarily positive. Treatment status for partially treated intervals is already coded using the midpoint approach. While a majority of partially treatment intervals were associated with a relative time of “zero,” some partially treated intervals for both PSL and PFL treatments had negative and positive values owing to variation in interval durations. In the main analysis, these cases were retained “as is” to ensure consistency between the DD and event study estimations. Relative treatment times for respondent intervals associated with untreated locations (non-paid leave mandate locations) were coded as zero throughout.

*Alternative Coding for Partially Treated Intervals*

Our data also includes partially treated intervals. These intervals contain the policy event and comprise some months that precede, and others that follow, the policy change. The annualized measure of relative time for a majority of partially treated intervals is positive or zero. In such cases, the partially treated intervals are coded as “treated.” In a few instances, the policy event comes towards the end of the interval, which leads to a negative value of relative time. This renders the partially treated interval as “untreated.” In Figure A1.1, suppose a policy event occurred in month 27 (two months before the interval ends). The difference between interval mid-point and policy event is (negative) nine months. As a result, the annualized relative time (rounded to nearest integer) is “-1” and the partially treated interval is coded as “pre-treatment” despite including the policy event and at least two treated months. In our main approach, we retain these cases “as is” to maintain consistency with the DD and event study analyses.

We considered three alternative approaches for handling partially treated intervals in our sensitivity tests. First, we assigned all partially treated intervals as “treated” irrespective of when during the interval the policy event took place. Second, we dropped partially treated intervals with negative values of relative time. Third, we estimated a model where we assigned each partially treated interval (for both PFL and PSL) a fractional treatment status given by the number of treated months (numerator) as a proportion of the total number of months in the interval (denominator). Pre- and post-treatment intervals associated with paid leave mandate location continued to be coded using a binary variable, and all intervals not associated with paid leave mandate location continued to be coded as zero. The third approach does not use the interval midpoint for assigning treatment status to partially treated intervals. Findings from sensitivity analyses are provided in Table A4 of the Supplementary Material.

.

Supplementary Material A2: Definitions and Coding of Outcome Variables and Covariates

*Outcome variable*

HRS respondents are coded as providers of personal care if they respond affirmatively to questions of the following form (note: question wording for the 2020 interview is shown; bracketed and parenthesized text reflects the fact that computer-assisted interviewing is used to tailor questions to the respondent’s situation regarding whether or not a spouse, mother, or father is recently deceased):

Did you [or your (late) [husband/wife/partner]] spend a total of 100 or more hours [since R's LAST IW MONTH, YEAR/in the last two years] helping your (deceased) [parents/mother/mother (and/or her husband)/father/father (and/or his wife)] with basic personal activities like dressing, eating, and bathing?

Provision of personal care is generated as binary outcomes and assessed at the end of the interval (given the retrospective nature of the question). Using data from the interview associated with the end of an interval, *personal care provision to parent* is coded as “1” if the respondent answers yes to the above question, and “0” otherwise.

*Individual-level covariates*

Coding of a parent’s need for care used the following questions:

Does [your mother] need help with basic personal needs like dressing, eating, or bathing?

Can [your mother] be left alone for an hour or more?

Has a doctor ever told your mother that she has Alzheimer’s Disease?

Has a doctor ever told your mother that she has dementia, senility or any other serious memory impairment?

Prior to 2020, the preceding two items were represented by a single question:

Has a doctor ever said that your mother has a memory-related disease?

The same questions are repeated for respondent’s father. *Parent care need* was coded as “1” if the respondent said “yes” in response to any of the questions above (at the beginning or the end of the interval) for either mother or father OR if a parent died during an interval, and “0” otherwise.

All other individual-level covariates are taken directly from HRS survey data elements (assessed in the baseline wave). These include:

Mother’s marital status (1: partnered/ 0: unpartnered)

Father’s marital status (1: partnered/ 0: unpartnered)

Respondent’s age (years)

Respondent’s marital status: (1: partnered/ 0: unpartnered)

Respondent: number of living brothers

Respondent: number of living sisters

Respondent: number of children under age 18 in the household

Respondent: Categorical variable for # of Activities of Daily Living (0-5) the respondent reported difficulty completing

In some specifications, we include a control for respondent’s apparent eligibility for the Family and Medical Leave Act (FMLA) program. Based on the federal criteria, FMLA eligibility is coded as “1” if a respondent reports:

- 12 months of job-tenure at current job
- 1,250 hours or greater worked over the past year (calculated based on weekly hours worked and total number of weeks worked in the past year)
- Firm size of 50 employees or greater

FMLA eligibility is coded as “0” if either of the above criteria are unmet or if respondent reports not working or self-employment.

*State-level covariates*

Minimum wage (CPI-adjusted US $): State minimum wage data compiled by University of Kentucky Center for Poverty Research [University of Kentucky Center for Poverty Research. (2023, Feb.). UKCPR National Welfare Data, 1980-2021. Lexington, KY. Available at http://ukcpr.org/resources/national-welfare-data (accessed March 29, 2023)]

Unemployment rate (%): State unemployment estimates compiled by University of Kentucky Center for Poverty Research [University of Kentucky Center for Poverty Research. (2023, Feb.). UKCPR National Welfare Data, 1980-2021. Lexington, KY. Available at http://ukcpr.org/resources/national-welfare-data (accessed March 29, 2023)]

Poverty Rate (%)**:** State poverty rate compiled by University of Kentucky Center for Poverty Research [University of Kentucky Center for Poverty Research. (2023, Feb.). UKCPR National Welfare Data, 1980-2021. Lexington, KY. Available at http://ukcpr.org/resources/national-welfare-data (accessed March 29, 2023)]

Affordable Care Act expansion (0/1): Status of ACA expansion across states compiled by Kaiser Family Foundation [Kaiser Family Foundation (2023, Mar.) Status of State Medicaid Expansion Decisions 2014-2022. Available at <https://www.kff.org/medicaid/issue-brief/status-of-state-medicaid-expansion-decisions-interactive-map/> (accessed March 29, 2023)]

Medicaid PCS State Plan Option (0/1)**:** Presence/Absence of personal care services as a Medicaid State Plan option

Pre-2005: LeBlanc, A., Tonner, M., & Harrington, C. (2001). State Medicaid programs offering personal care services. Health Care Financing Review, 22, 155–173.

2005-2009: Kaiser Commission on Medicaid and the Uninsured. (2005 to 2009). Medicaid 1915(c) home and community-based service programs: Data update (Issue Paper).

2009-2018: Annual Medicaid LTSS expenditure report prepared for the Centers for Medicare & Medicaid Services (CMS) by *Truven Analytics* and *Mathematica* from 2008–2018.

Eiken S, Sredl K, Gold L, et al. Medicaid Expenditures for Long-Term Services and Supports in FFY 2012. Centers for Medicare & Medicaid Services. Mathematica Policy Research Institute. Truven Health Analytics. Accessed July 29, 2021. http://www.advancingstates.org/sites/nasuad/ files/LTSS_Expenditures_2012.pdf 35.

Eiken S, Sredl K, Burwell B, Saucier P. Medicaid Expenditures for Long-Term Services and Supports (LTSS) in FY 2013: Home and Community-Based Services were a Majority of LTSS Spending. 2015. Centers for Medicare & Medicaid Services. Accessed July 29, 2021. https:// www.medicaid.gov/sites/default/files/2019-12/ltss-expendituresfy2013final.pdf 36.

Eiken S, Sredl K, Burwell B, Woodward R. Medicaid Expenditures for Long-Term Services and Supports (LTSS) in FY 2015. 2017. Centers for Medicare & Medicaid Services. Accessed July 29, 2021. https://www.medicaid. gov/sites/default/files/2019-12/ltssexpendituresffy2015final.pdf

Murray, C., Tourtellotte, A. Lipson, D, Wysocki, A. Medicaid Long Term Services and Supports Annual Expenditures Report: Federal Fiscal Years 2017 and 2018. Chicago, IL: Mathematica, January 7, 2021. Accessed March 29, 2023. https://www.medicaid.gov/medicaid/long-term-services-supports/downloads/ltssexpenditures-2017-2018.pdf

Supplementary Material A3: Full regression results

| **Table A3.1: Changes in probability of personal care provision to parents associated with PFL & PSL mandates** | | | | | |  |
| --- | --- | --- | --- | --- | --- | --- |
|  |  |  |  |  |  |  |
|  |  |  |  |  |  |  |
|  | (1) | (2) | (3) | (4) | (5) |  |
|  | Full sample, no covariates | Full sample, with covariates | Baseline workers, with covariates | Full sample, all covariates (incl. FMLA eligibility) | Baseline workers, all covariates (incl. FMLA eligibility) |  |
|  |  |  |  |  |  |  |
|  |  |  |  |  |  |  |
| PFL mandate | -0.002 | -0.001 | -0.014 | 0.014 | 0.008 |  |
|  | (0.018) | (0.013) | (0.027) | (0.014) | (0.030) |  |
| PSL mandate | 0.041** | 0.047** | 0.053** | 0.046** | 0.053* |  |
|  | (0.014) | (0.014) | (0.019) | (0.015) | (0.021) |  |
| FMLA eligibility |  |  |  | -0.014 | -0.004 |  |
|  |  |  |  | (0.009) | (0.011) |  |
| Parent care need |  | 0.145*** | 0.125*** | 0.145*** | 0.123*** |  |
|  |  | (0.007) | (0.009) | (0.007) | (0.009) |  |
| Mom partnered |  | -0.013 | -0.007 | -0.019 | -0.011 |  |
|  |  | (0.017) | (0.021) | (0.019) | (0.024) |  |
| Dad partnered |  | -0.014 | -0.014 | -0.013 | -0.0191 |  |
|  |  | (0.019) | (0.022) | (0.019) | (0.027) |  |
| Age |  | -0.005 | -0.006 | -0.008 | -0.012 |  |
|  |  | (0.004) | (0.005) | (0.004) | (0.007) |  |
| Living brothers |  | -0.004 | -0.004 | -0.006 | -0.008 |  |
|  |  | (0.006) | (0.009) | (0.007) | (0.012) |  |
| Living sisters |  | -0.009 | -0.006 | -0.008 | -0.003 |  |
|  |  | (0.006) | (0.009) | (0.007) | (0.010) |  |
| Number of children |  | 0.001 | 0.003 | 0.001 | -0.001 |  |
|  |  | (0.005) | (0.005) | (0.006) | (0.005) |  |
| Partnered |  | -0.016 | -0.030 | -0.019 | -0.031 |  |
|  |  | (0.012) | (0.016) | (0.012) | (0.016) |  |
| Respondent ADLs |  |  |  |  |  |  |
| 0 |  | reference | reference | reference | reference |  |
|  |  |  |  |  |  |  |
| 1 |  | -0.019* | -0.027 | -0.024* | -0.039* |  |
|  |  | (0.009) | (0.016) | (0.009) | (0.019) |  |
| 2 |  | -0.004 | 0.027 | -0.008 | 0.035 |  |
|  |  | (0.018) | (0.040) | (0.017) | (0.042) |  |
| 3 |  | -0.048* | 0.070 | -0.052* | 0.105 |  |
|  |  | (0.022) | (0.055) | (0.022) | (0.055) |  |
| 4 |  | -0.037 | 0.143 | -0.042 | 0.160 |  |
|  |  | (0.025) | (0.083) | (0.026) | (0.100) |  |
| 5 |  | -0.013 | 0.021 | -0.011 | 0.033 |  |
|  |  | (0.035) | (0.024) | (0.037) | (0.019) |  |
| Minimum wage |  | 0.001 | -0.002 | 0.001 | -0.005 |  |
|  |  | (0.006) | (0.006) | (0.005) | (0.006) |  |
| Unemployment rate |  | -0.001 | -0.006 | -0.001 | -0.007 |  |
|  |  | (0.003) | (0.005) | (0.004) | (0.005) |  |
| Poverty rate |  | 0.003 | 0.002 | 0.003 | 0.001 |  |
|  |  | (0.002) | (0.002) | (0.002) | (0.003) |  |
| ACA expansion |  | -0.007 | -0.013 | -0.006 | -0.011 |  |
|  |  | (0.013) | (0.018) | (0.015) | (0.020) |  |
| Medicaid PCS |  | 0.025* | 0.020 | 0.020 | 0.015 |  |
|  |  | (0.011) | (0.013) | (0.013) | (0.013) |  |
| N | 40,077 | 38,183 | 23,338 | 34,818 | 20,074 |  |
| Notes: Estimations restricted to respondents age 50 and older and to those with at least one parent alive at baseline. All regressions control for individual, area, and year fixed effects. *C*lustered (state-level) standard errors in parentheses. * *p*<0.05, ** *p*<0.01, *** *p*<0.001 | | | | | |  |
|  |  |  |  |  |  |  |
|  |  |  |  |  |  |  |
|  |  |  |  |  |  |  |

| **Table A3.2: Changes in probability of personal care provision to parents associated with different types of paid leave exposure** | | | | |  |
| --- | --- | --- | --- | --- | --- |
|  |  |  |  |  |  |
|  |  |  |  |  |  |
|  | (1) | (2) | (4) | (5) |  |
|  | Full sample, with covariates | Baseline workers, with covariates | Full sample, all covariates (incl. FMLA eligibility) | Baseline workers, all covariates (incl. FMLA eligibility) |  |
|  |  |  |  |  |  |
|  |  |  |  |  |  |
| PFL NJP Only | -0.010 | -0.036 | 0.004 | -0.018 |  |
|  | (0.009) | (0.019) | (0.008) | (0.019) |  |
| PFLJP Only | 0.014 | 0.037* | 0.035* | 0.057** |  |
|  | (0.013) | (0.016) | (0.013) | (0.017) |  |
| PSL Only | 0.034 | 0.036 | 0.030 | 0.027 |  |
|  | (0.020) | (0.027) | (0.019) | (0.022) |  |
| PSL-PFLJP | 0.065*** | 0.068** | 0.076*** | 0.099*** |  |
|  | (0.017) | (0.021) | (0.018) | (0.023) |  |
| PSL-PFLNJP | 0.052** | 0.039* | 0.067*** | 0.063** |  |
|  | (0.016) | (0.019) | (0.016) | (0.021) |  |
| FMLA eligibility |  |  | -0.015 | -0.004 |  |
|  |  |  | (0.009) | (0.011) |  |
| Parent care need | 0.145*** | 0.125*** | 0.145*** | 0.123*** |  |
|  | (0.007) | (0.009) | (0.007) | (0.009) |  |
| Mom partnered | -0.014 | -0.008 | -0.019 | -0.011 |  |
|  | (0.018) | (0.021) | (0.018) | (0.024) |  |
| Dad partnered | -0.014 | -0.014 | -0.013 | -0.0191 |  |
|  | (0.019) | (0.022) | (0.020) | (0.027) |  |
| Age | -0.005 | -0.006 | -0.008 | -0.012 |  |
|  | (0.004) | (0.005) | (0.004) | (0.007) |  |
| Living brothers | -0.004 | -0.004 | -0.006 | -0.008 |  |
|  | (0.006) | (0.009) | (0.007) | (0.012) |  |
| Living sisters | -0.009 | -0.005 | -0.008 | -0.003 |  |
|  | (0.006) | (0.009) | (0.007) | (0.010) |  |
| Number of children | 0.001 | 0.002 | 0.001 | -0.001 |  |
|  | (0.005) | (0.005) | (0.006) | (0.005) |  |
| Partnered | -0.016 | -0.030 | -0.019 | -0.030 |  |
|  | (0.012) | (0.016) | (0.012) | (0.015) |  |
| Respondent ADLs |  |  |  |  |  |
| 0 | reference | reference | reference | reference |  |
|  |  |  |  |  |  |
| 1 | -0.019* | -0.028 | -0.024* | -0.039* |  |
|  | (0.009) | (0.016) | (0.009) | (0.019) |  |
| 2 | -0.004 | 0.027 | -0.008 | 0.035 |  |
|  | (0.018) | (0.040) | (0.017) | (0.042) |  |
| 3 | -0.047* | 0.070 | -0.052* | 0.105 |  |
|  | (0.022) | (0.055) | (0.022) | (0.055) |  |
| 4 | -0.037 | 0.144 | -0.042 | 0.161 |  |
|  | (0.025) | (0.083) | (0.026) | (0.100) |  |
| 5 | -0.012 | 0.021 | -0.011 | 0.034 |  |
|  | (0.035) | (0.024) | (0.036) | (0.019) |  |
| Minimum wage | 0.0004 | -0.004 | 0.001 | -0.005 |  |
|  | (0.006) | (0.006) | (0.005) | (0.006) |  |
| Unemployment rate | -0.001 | -0.006 | -0.001 | -0.007 |  |
|  | (0.003) | (0.005) | (0.005) | (0.006) |  |
| Poverty rate | 0.003 | 0.001 | 0.002 | 0.0003 |  |
|  | (0.002) | (0.003) | (0.002) | (0.003) |  |
| ACA expansion | -0.008 | -0.016 | -0.007 | -0.015 |  |
|  | (0.013) | (0.018) | (0.014) | (0.020) |  |
| Medicaid PCS | 0.024* | 0.019 | 0.020 | 0.014 |  |
|  | (0.011) | (0.013) | (0.012) | (0.012) |  |
| N | 40,077 | 38,183 | 34,818 | 20,074 |  |
| Notes: PFLJP: Job Protected PFL; PFLNJP: Non-Job Protected PFL. Estimations restricted to respondents age 50 and older and to those with at least one parent alive at baseline. All regressions control for individual, area, and year fixed effects. *C*lustered (state-level) standard errors in parentheses. * *p*<0.05, ** *p*<0.01, *** *p*<0.001 | | | | |  |
|  |  |  |  |  |  |
|  |  |  |  |  |  |
|  |  |  |  |  |  |
|  |  |  |  |  |  |

Supplementary Material A4: Sensitivity Tests

| **Table A4: Sensitivity Tests** | | | | | | | |  |
| --- | --- | --- | --- | --- | --- | --- | --- | --- |
|  |  |  |  |  |  |  |  |  |
|  | (1) | (2) | (3) | (4) | (5) | (6) | (7) |  |
|  | Non-movers | Separate treatments | | Partially Treated Interval (PTI) coding | | | 2020 dropped |  |
|  |  | No PSL control | No PFL control | All PTI treated | Negative PTI dropped | Fractional treatment |  |  |
|  |  |  |  |  |  |  |  |  |
| PFL mandate | 0.004 | -0.001 |  | -0.001 | -0.009 | -0.012 | -0.009 |  |
|  | (0.011) | (0.012) |  | (0.012) | (0.013) | (0.012) | (0.011) |  |
| PSL mandate | 0.048*** |  | 0.047** | 0.042* | 0.047** | 0.049** | 0.056*** |  |
|  | (0.013) |  | (0.014) | (0.016) | (0.015) | (0.016) | (0.014) |  |
| N | 34,963 | 38,183 | 38,183 | 38,183 | 37,751 | 38,183 | 34,259 |  |
| *Notes*: Estimations restricted to respondents aged 50 years and older and to those with at least one parent alive at baseline. Sample in Model 1 restricted to respondents with the same location ID within the panel (i.e., non-movers). Covariates in all models include: parent care need, mother's partnered status, father's partnered status, respondent age, number of brothers, number of sisters, number of children, respondent ADLs, Minimum Wage, Unemployment Rate, Poverty Rate, Medicaid ACA Expansion status, and the presence of Medicaid Personal Care Services State Plan Option. Model 1 controls for individual and year fixed effects. Models 2-7 control for individual, location, and year fixed effects. Clustered (state-level) standard errors in parentheses. * p<0.05, ** p<0.01, *** p<0.001 | | | | | | | |  |
|  |  |  |  |  |  |  |  |  |
|  |  |  |  |  |  |  |  |  |
|  |  |  |  |  |  |  |  |  |
